# Eph/Ephrin Profiling in Human Breast Cancer Reveals Significant Associations between Expression Level and Clinical Outcome

**DOI:** 10.1371/journal.pone.0024426

**Published:** 2011-09-15

**Authors:** Dana M. Brantley-Sieders, Aixiang Jiang, Krishna Sarma, Akosua Badu-Nkansah, Debra L. Walter, Yu Shyr, Jin Chen

**Affiliations:** 1 Veterans Affairs Medical Center, Tennessee Valley Healthcare System, Nashville, Tennessee, United States of America; 2 Department of Medicine, Vanderbilt University School of Medicine, Nashville, Tennessee, United State of America; 3 Department of Cell and Developmental Biology, Vanderbilt University School of Medicine, Nashville, Tennessee, United States of America; 4 Department of Cancer Biology, Vanderbilt University School of Medicine, Nashville, Tennessee, United States of America; 5 Vanderbilt-Ingram Comprehensive Cancer Center, Vanderbilt University School of Medicine, Nashville, Tennessee, United States of America; 6 Department of Biostatistics, Vanderbilt University School of Medicine, Nashville, Tennessee, United States of America; 7 Department of Biochemistry, Vanderbilt University School of Medicine, Nashville, Tennessee, United States of America; University of Pennsylvania School of Medicine, United States of America

## Abstract

Pre-clinical studies provide compelling evidence that Eph family receptor tyrosine kinases (RTKs) and ligands promote cancer growth, neovascularization, invasion, and metastasis. Tumor suppressive roles have also been reported for the receptors, however, creating a potential barrier for clinical application. Determining how these observations relate to clinical outcome is a crucial step for translating the biological and mechanistic data into new molecularly targeted therapies. We investigated *eph* and *ephrin* expression in human breast cancer relative to endpoints of overall and/or recurrence-free survival in large microarray datasets. We also investigated protein expression in commercial human breast tissue microarrays (TMA) and Stage I prognostic TMAs linked to recurrence outcome data. We found significant correlations between *ephA2*, *ephA4*, *ephA7*, *ephB4*, and *ephB6* and overall and/or recurrence-free survival in large microarray datasets. Protein expression in TMAs supported these trends. While observed no correlation between *ephrin* ligand expression and clinical outcome in microarray datasets, ephrin-A1 and EphA2 protein co-expression was significantly associated with recurrence in Stage I prognostic breast cancer TMAs. Our data suggest that several Eph family members are clinically relevant and tractable targets for intervention in human breast cancer. Moreover, profiling Eph receptor expression patterns in the context of relevant ligands and in the context of stage may be valuable in terms of diagnostics and treatment.

## Introduction

According to the American Cancer Society, 207,090 new cases of invasive breast cancer were anticipated for women in the U.S. during 2010. Breast cancer is the second most frequently diagnosed cancer in U.S. women, predicted to result in 39,840 deaths in 2010, and ranks second as a cause of cancer death in women (ACS, Breast Cancer Facts and Figures 2010 Atlanta, GA). Understanding the molecular mechanisms that regulate progression of this devastating disease is crucial for identifying novel therapeutic targets. Current treatment options, such as adjuvant chemotherapy or radiation, have improved survival, particularly in women diagnosed with early stage breast cancer (ACS, Breast Cancer Facts and Figures 2010 Atlanta, GA). Existing treatments, however, are often accompanied by undesirable side effects that significantly reduce patient quality of life (e.g. gastrointestinal discomfort, lymphedema, menopausal-like symptoms/premature menopause, impaired cognitive function/neurotoxicity, adverse physical and psychological effects on sexuality) and/or increase the risk of mortality [e.g. cardiac toxicity, increased risk for secondary cancers, bone loss; reviewed in [1], [2], [3], [4]]. One of the proposed benefits for new, molecularly targeted therapies is the potential to reduce morbidity associated with cancer as well as mortality.

Several pre-clinical and laboratory studies support the function of Eph receptor tyrosine kinases (RTKs) in tumor growth, metastasis, and neovascularization [reviewed in [5], [6], [7]], including breast cancer [reviewed in [8]]. The Eph family of RTKs is the largest identified in the vertebrate genome and is subdivided into class A and class B based on sequence homology and binding affinity for two distinct types of membrane-anchored ephrin ligands. Class A receptors normally interact with glycosyl-phosphatidylinositol (GPI)-linked class A ephrins, while class B receptors generally bind to class B ephrins that are attached to the cell membrane by a transmembrane-spanning domain, although interclass binding does occur among certain family members [5]. Originally characterized as axon guidance regulators, ephrins and Eph RTKs regulate physiologic and pathologic processes during embryonic development, in normal tissue homeostasis, and in disease [reviewed in [5], [6], [9]], making them attractive candidates for new molecularly targeted therapies, particularly in cancer. However, with 14 receptors (9 class A and 5 class B) and 8 ligands (5 class A and 3 class B) present in the human genome, expression patterns that often overlap, and promiscuous interaction between ligands and receptors that include bi-directional signaling and pleiotropic functions, the role of Eph receptors in cancer is extremely complex [5]. Moreover, the role of Eph and ephrin molecules in tumor progression remains controversial, with evidence suggesting both tumor promoting and tumor suppressive functions [reviewed in [5], [8]].

We sought to address this controversy by profiling expression of Eph RTKs and ephrin ligands in human breast cancer. We compared mRNA expression levels with clinical outcome in human breast cancer microarray datasets, as well as protein expression in tumor epithelium in human breast cancer tissue microarrays. These analyses confirmed the relevance of EphA2 and EphB4 to human breast cancer progression, and uncovered significant correlations for EphA4, EphA7, and EphB6, which were previously under-investigated in breast cancer. Coupled with human breast cancer TMAs for which clinical data were available, our data suggest that several Eph family members are clinically relevant and tractable targets for intervention in human breast cancer. Our data further suggest that profiling Eph RTK expression in the context of ligand expression and stage may prove more informative in terms of diagnostics and patient selection for molecularly targeted therapy trials using anti-Eph RTK agents.

## Results

### Elevated RNA expression of many Eph RTKs significantly correlates with poor outcome in human breast cancer

**Figure 1:**
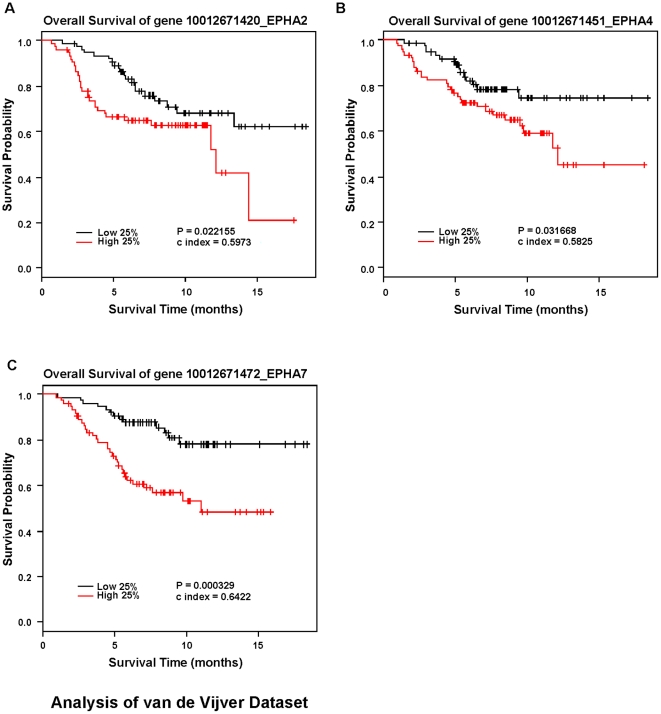
RNA expression of EphA2, EphA4, and EphA7 negatively correlates with overall survival in human breast cancer. Kaplan-Meier kinetic analyses of the van der Vijver dataset, with microarray profiles of 295 human breast tumors and associated clinical data. The impact of elevated *ephA2* (A), *ephA4* (B), and *ephA7* (C), expression on overall survival was analyzed by Log-Rank tests.

**Figure 2:**
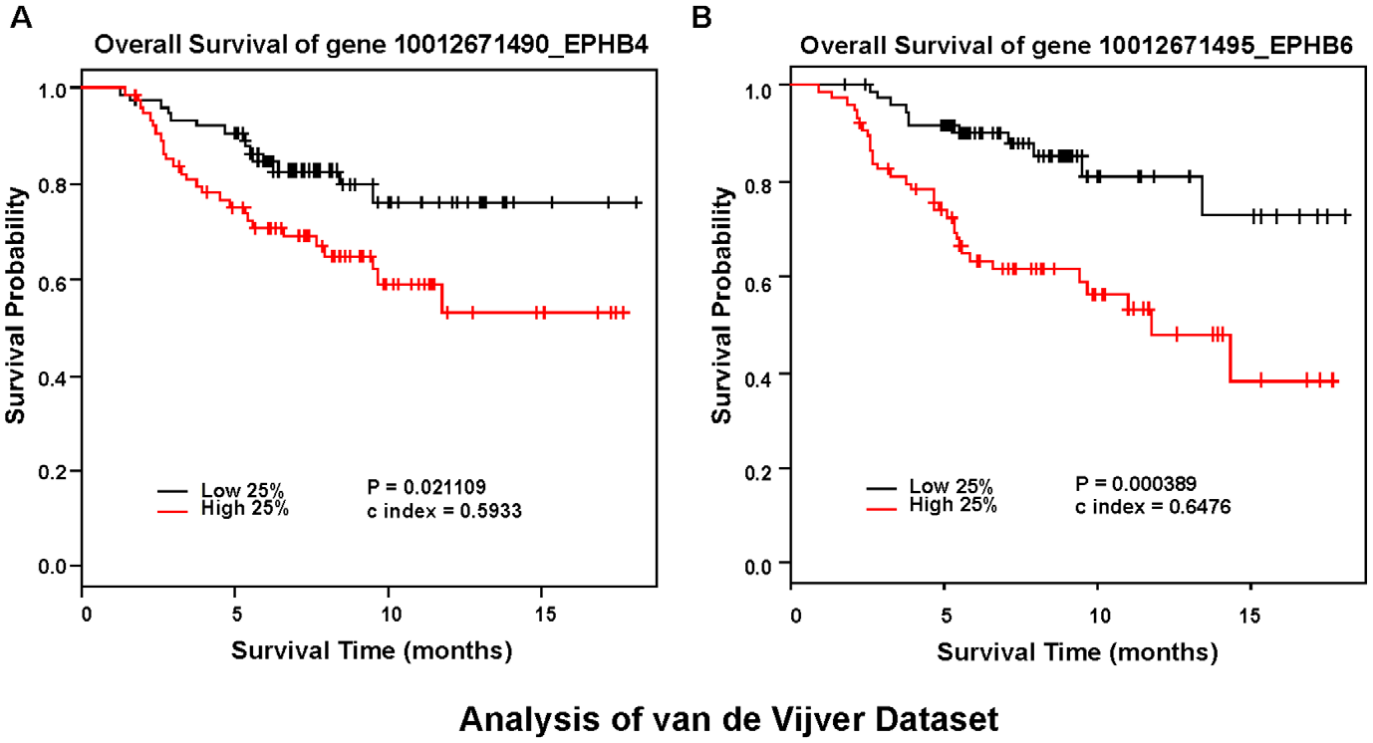
RNA expression of EphB4 and EphB6 negatively correlates with overall survival in human breast cancer. Kaplan-Meier kinetic analyses of the van der Vijver dataset, with microarray profiles of 295 human breast tumors and associated clinical data. The impact of elevated *ephB4* (A) and *ephB6* (B) expression on overall survival was analyzed by Log-Rank tests.

Several reports in the literature provide evidence that individual Eph receptors contribute to breast tumorigenesis and progression [reviewed in [8]]. The clinical relevance of these observations, however, remains under-investigated. Moreover, expression patterns for several members of this large RTK family have yet to be determined in cancer. To address these gaps in our knowledge, we profiled the expression of individual Eph and ephrin family members in relation to overall and/or recurrence free survival in two independent breast cancer patient datasets [10], [11]. In the van de Vijver dataset, consisting of 295 patient samples, relatively high RNA expression levels of *ephA2*, *ephA4*, *ephA7*, *ephB4*, and *ephB6* correlated significantly with reduced overall survival (Figures 1&2). Similar trends were observed for recurrence-free survival in this dataset (Figure S1&2), as well as metastasis-free survival in the independent van't Veer dataset that consisted of 117 patient samples (Figures S3&S4). We did not observe any positive or negative associations between expression level and clinical outcome for other Eph RTK family members analyzed, nor did we observe any significant associations between any ephrin ligand family member expression and outcome (data not shown; note that *ephA6* and *ephrin-A2* probes were not represented in these datasets). Analysis of estrogen and progesterone receptor RNA expression and clinical outcome were used as internal controls (Figure S5).

The Kaplan-Mier curves were generated using relative expression data from the top and bottom quartiles of high and low expression. When we analyzed *ephA2*, *ephA4*, *ephA7*, *ephB4*, and *ephB6* as continuous covariates using the entire dataset, Cox model analysis supported the observed association between these receptor family members and overall survival in the van de Vijver dataset (Table 1) and metastasis-free survival in the Veer dataset (Table S1), with EphA2 expression showing the most consistent, significant association with poor outcome. Our analyses suggest that these specific Eph RTK family members are the most clinically relevant to breast cancer, making them attractive candidates for further analysis.

**Table 1:** Association between Eph receptor expression and overall survival in human breast cancer.

Eph Receptor	RR Overall Survival (95% CI)	P-Value
EphA2	8.52 (2.8–25.9)	0.0002*
EphA4	3.32 (1.49–7.39)	0.0003*
EphA7	3.55 (1.12–11.3)	0.0316*
EphB4	17.2 (1.91–155.1)	0.0111*

Cox Model Analysis: Relative risk (RR) associated with elevated Eph receptor molecule expression and overall survival in the van de Vijver dataset.

*Statistically significant association.

### Eph RTKs associated with poor clinical outcome are also overexpressed at the protein level in human breast cancer samples

**Figure 3:**
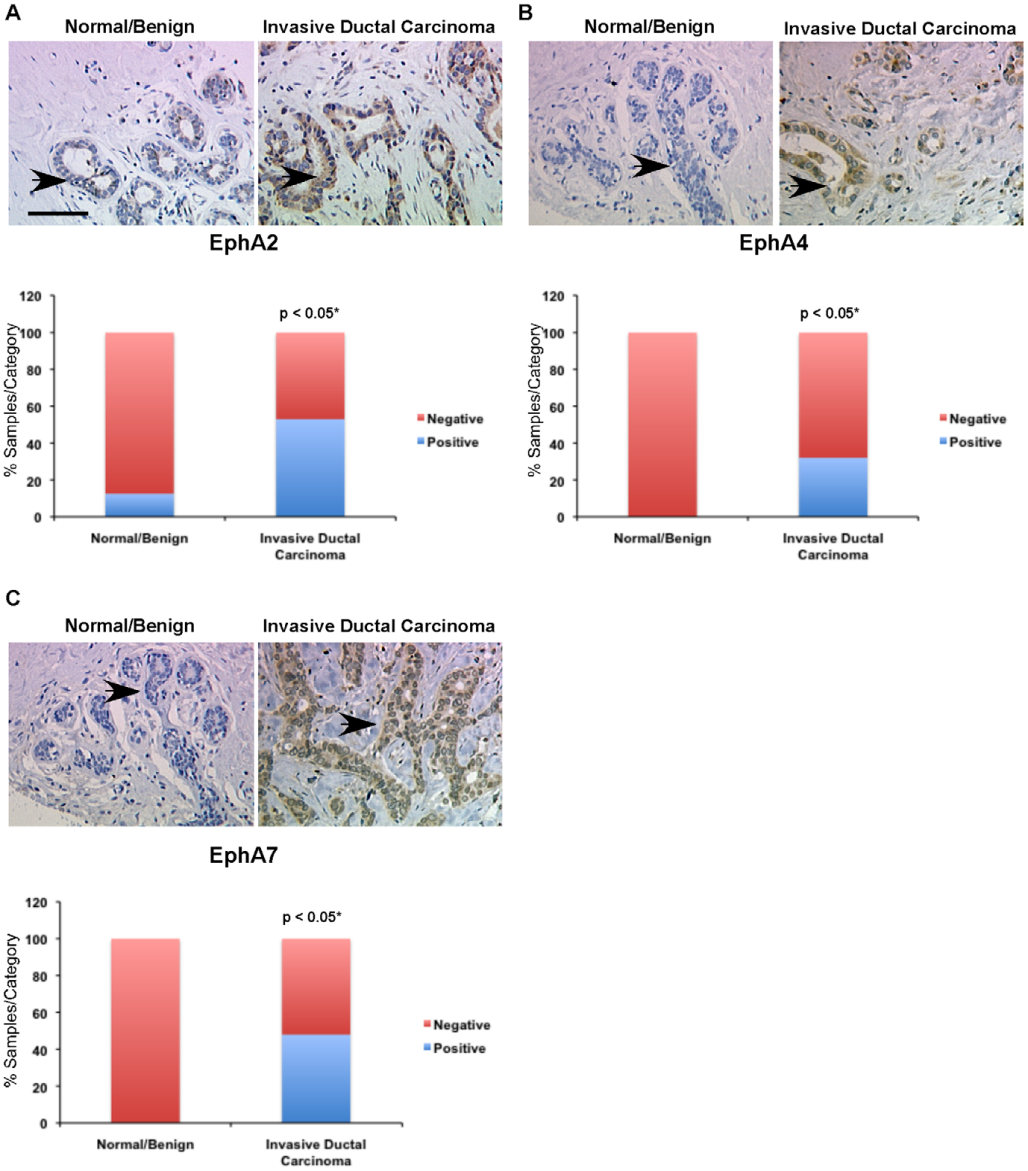
Protein expression of EphA2, EphA4, and EphA7 is elevated in human ductal carcinoma relative to normal/benign tissue controls. Immunohistochemical analysis of human breast tissue microarrays (TMAs) was performed to compare relative expression in normal/benign epithelium (n = 8 samples) versus invasive ductal carcinoma (n = 126 samples) for EphA2 (A), EphA4 (B), and EphA7 (C). Arrows indicate tumor epithelium in photomicrographs. Scale bar = 50 µm. *Differential expression between normal/benign and malignant epithelium was assessed using Chi Square analysis.

**Figure 4:**
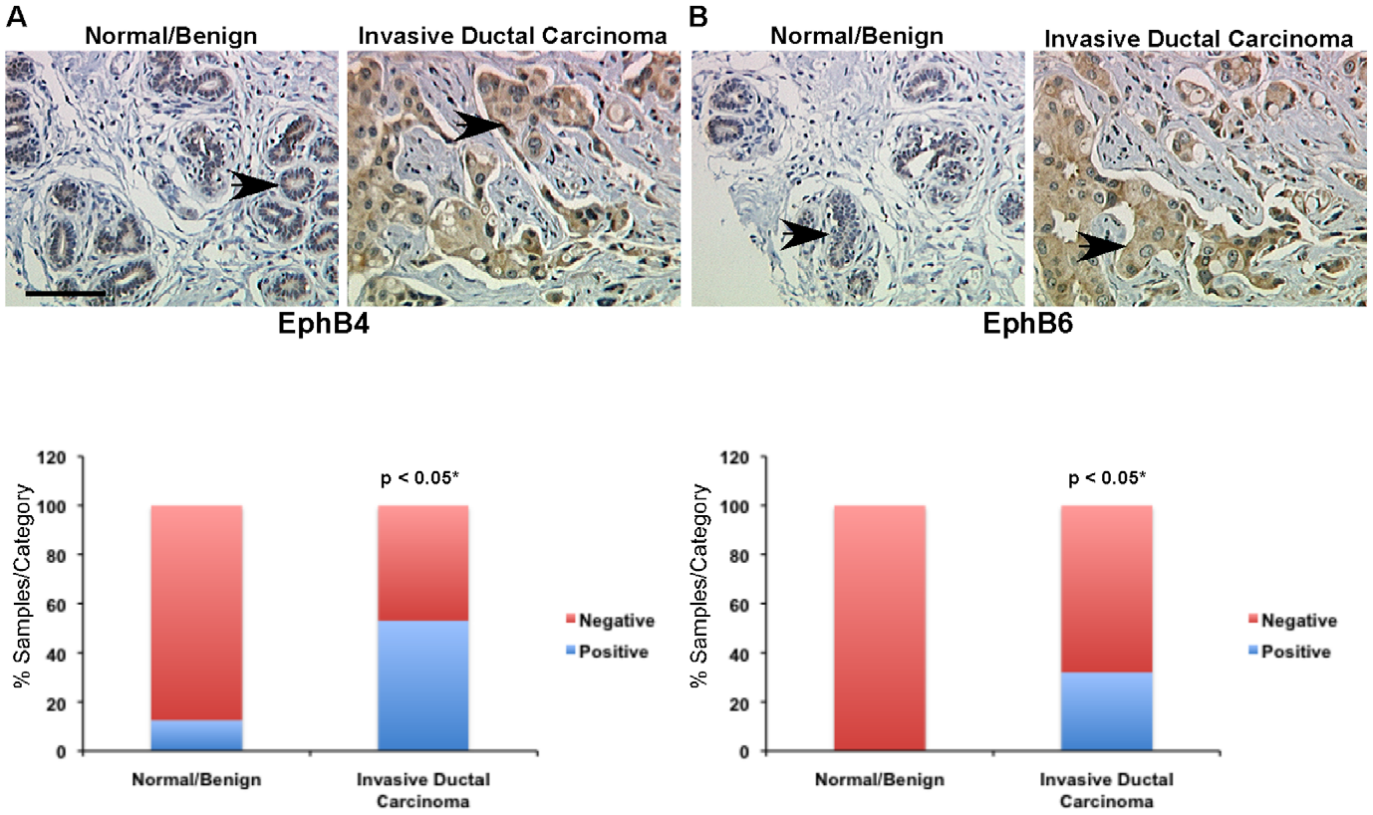
Protein expression of EphB4 and EphB6 is elevated in human ductal carcinoma relative to normal/benign tissue controls. Immunohistochemical analysis of human breast tissue microarrays (TMAs) was performed to compare relative expression in normal/benign epithelium (n = 8 samples) versus invasive ductal carcinoma (n = 126 samples) for EphB4 (A) and EphB6 (B). Arrows indicate tumor epithelium in photomicrographs. Scale bar = 50 µm. *Differential expression between normal/benign and malignant epithelium was assessed using Chi Square analysis.

We evaluated protein expression of EphA2, EphA4, EphA7, EphB4, and EphB6 in human breast cancer tissue microarrays (TMA) in which we could distinguish expression in tumor epithelium from stromal components, including endothelium. We compared expression in normal and hyperplastic breast tissue to levels observed in invasive ductal carcinoma using a continuous scale to rank relative expression in tumor epithelium based on the percentage of positive tumor epithelial cells within each core as well as relative intensity of staining. A significantly higher percentage of human invasive ductal carcinoma samples displayed expression of EphA2, EphA4, or EphA7 in tumor epithelium relative to ‘normal’ samples (normal/hyperplastic or fibroadenoma), which were largely negative (Figure 3). EphB4 and EphB6 were also significantly elevated in tumor epithelium relative to ‘normal’ samples (Figure 4). By contrast, EphA8, which was not significantly associated with clinical outcome in the microarray datasets, did not show significant elevation in human breast cancer tissue relative to controls (Figure S6). Antibody specificity was validated using tissue from targeted deletion mutant mice or peptide competition (Figure S7). Together, these data suggest that RNA expression profiles within the microarray datasets correlate with protein expression levels in tumor epithelium.

### Ephrin-A1 association with EphA2 and clinical outcome

Although we did not observe any significant associations between ephrin ligand expression and clinical outcome, several studies suggest that ephrins do play a role in breast tumor progression and angiogenesis [reviewed in [12]]. Ligand-dependent versus ligand-independent signaling has emerged as one key mechanism underlying the tumor suppressive functions as opposed to oncogenic effects of Eph RTKs. Thus, we analyzed co-expression of ephrin-A1, the primary ligand for EphA2, and EphA2 protein in a large Stage I prognostic TMA from the NCI Cooperative Breast Cancer Tissue Resource (CBCTR) Cancer Diagnosis Program (CDP). This TMA consists of over 500 breast cancer plus normal breast tissue samples that are linked to recurrence outcome data (http://cdp.nci.nih.gov/breast/prognostic_tma.html).

We stained duplicate slides with anti-ephrin-A1 or anti-EphA2 antibodies that were validated using mammary tissue from genetic deletion mouse models (Figure S7) and quantified the percent positive tumor epithelium and relative staining intensity using a computer-based Ariol platform. Expression of EphA2 was not statistically associated with recurrence, nor was there a correlation between ephrin-A1 and recurrence (data not shown). Pearson's product-moment correlation, however, revealed a statistically significant correlation (r = 0.4982) between ephrin-A1 and EphA2 coexpression when analyzed as continuous variables in the subset of 72 patients that displayed disease recurrence [p-value <0.0001; 95% CI = 0.2998, 0.6554].

The correlation between ephrin-A1 and EphA2 coexpression in the non-recurrent patients was completed using the bootstrap based correlation analysis. We randomly selected (with replacement) a sample size of 72 from 446 non-recurrent patients 10,000 times. The mean correlation between ephrin-A1 and EphA2 from the bootstrap analysis was 0.3475 (p-value >0,05), which was not statistically significant.

**Figure 5:**
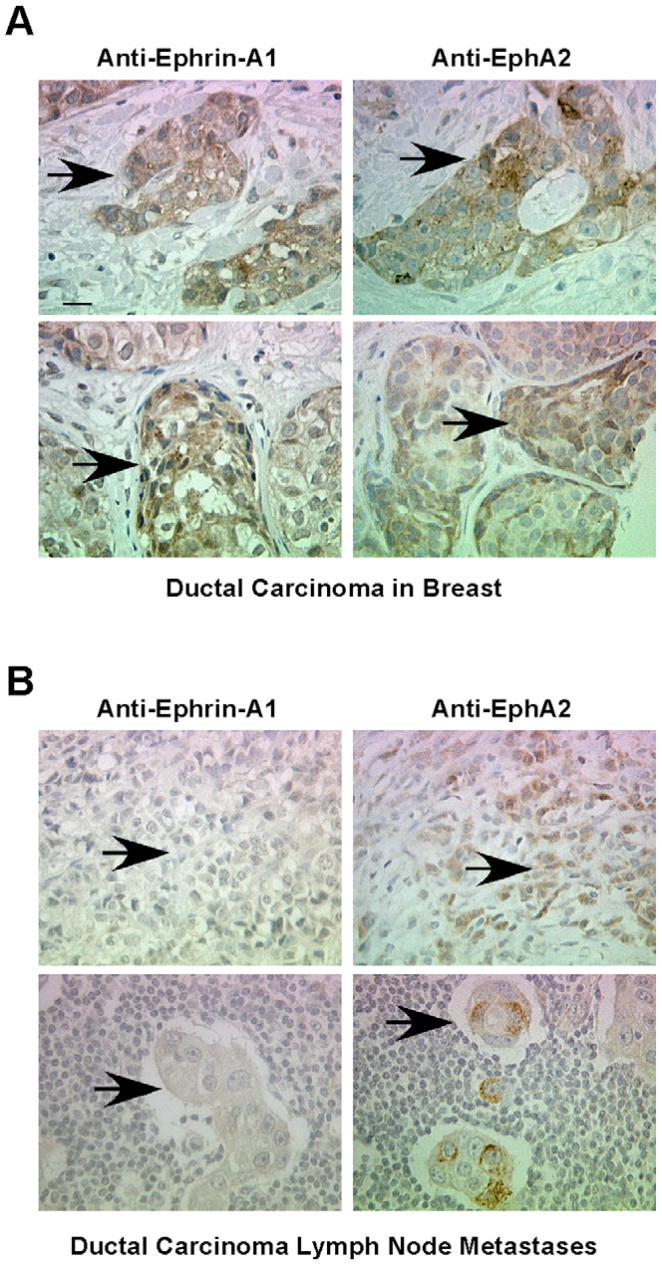
Inverse correlation between Ephrin-A1 and EphA2 expression in metastatic human breast cancer. Immunohistochemical staining was performed for human breast cancer samples on commercial TMAs. (A) We observed co-expression of EphA2 in several infiltrating ductal carcinoma samples confined to breast tissue. (B) In infiltrating ductal carcinomas that metastasized to the lymph node, however, we observed that a significant portion of samples showed mutually exclusive staining patterns for EphA2 and ephrin-A1 (p<0.05, Chi Square analysis; n = 32 total lymph node metastasis samples, 2 out of 32 ephrin-A1+/EphA2+ versus 9 out of 32 ephrin-A1+/EphA2− and 8 out of 32 ephrin-A1−/EphA2+). In the subset of samples that were ephrin-A1+ (11 out of 32), 2 out of 11 were ephrin-A1+/EphA2+ versus 9 out of 11 ephrin-A1+/EphA2− (p<0.05, Chi Square analysis). Arrowheads indicate metastatic tumor cells embedded within lymph node tissue. Scale bar = 25 µm.

These data were consistent with our observations in commercial TMAs in which EphA2 and ephrin-A1 proteins were co-expressed in ductal carcinoma samples confined to the breast (Figure 5). In infiltrating ductal carcinoma samples that metastasized to lymph node, however, we observed that a significant portion of the samples displayed mutually exclusive staining patterns for EphA2 and ephrin-A1 (p<0.05, Chi square analysis; n = 32 total lymph node metastastic samples, 2 ephrin-A1+/EphA2+ versus 9 ephrin-A1+/EphA2− and 8 ephrin-A1−/EphA2+). In the subset of samples that were ephrin-A1+ (11 out of 32), 2 were ephrin-A1+/EphA2+ and 9 were ephrin-A1+/EphA2− (p<0.05, Chi square analysis). Together, these data suggest that while co-expression of receptor and ligand in early stage breast cancer may contribute to recurrence, loss of ephrin-A1 ligand in metastatic samples may contribute to invasion, as suggested by laboratory studies [reviewed in [5], [12]].

## Discussion

Studies using both murine and human cancer cell lines in culture and in allograft/xenograft models, as well transgenic and gene-deletion mouse models, provide strong evidence that several members of the Eph family of receptor tyrosine kinases and their ephrin ligands regulate tumorigenesis and progression [reviewed in [5]]. Although some previous studies included expression analysis in patient samples, large-scale expression profiling for these molecule in relation to clinical outcome has been limited, including profiling in breast cancer. In this study, we analyzed large microarray datasets for human breast cancer samples linked to clinical data, as well as human breast tissue microarrays, to identify associations between specific Eph and ephrin family members and overall survival and/or recurrence. We observed a significant negative association between expression of EphA2 and EphB4 and outcome, consistent with previous preclinical studies. In addition, we uncovered previously uncharacterized associations between expression of EphA4, EphA7, and EphB6 and survival/recurrence, highlighting the value of this strategy in uncovering novel, clinically relevant targets. Although we did not observe any significant associations with ephrin ligand expression and clinical outcome, co-expression of EphA2 and ephrin-A1 in Stage I breast cancers correlated with recurrence. By contrast, a disproportionate number of metastatic ductal carcinoma samples that were EphA2 positive displayed little or no ephrin-A1 expression. These data suggest that profiling Eph receptor expression patterns in the context of relevant ligands may be valuable in terms of diagnostics.

Of the A class Eph RTKs, EphA2 is most extensively studied in breast cancer [Reviewed in [5], [8]]. Our profiling data are consistent with previous studies reporting that EphA2 is expressed at low levels in normal breast epithelium [13], [14] and overexpressed in 60–80% of breast cancers [15], [16], [17]. Forced overexpression of EphA2 resulted in malignant transformation of non-transformed MCF10A breast cells [16], whereas Conversely, siRNA-mediated inhibition of EphA2 or overexpression of dominant-negative EphA2 constructs suppressed growth and metastasis of MMTV-Neu tumor cells and 4T1 metastatic mouse mammary adenocarcinoma cells, respectively, *in vivo* [18], [19]. Targeted disruption of EphA2 also impaired normal mammary epithelial growth and branching morphogenesis [12], as well as tumor initiation and lung metastasis in the MMTV-Neu transgenic model of mammary adenocarcinoma [18]. More recently, EphA2 was reported to mediate resistance to trastuzumab therapy [20], and to affect estrogen dependence and tamoxifen sensitivity [21], in cell line and xenograft models.

Similarly, EphB4 levels are also elevated in human breast cancer [22]. EphB4 knockdown inhibited breast cancer survival, migration, and invasion in vitro and tumor growth in a xenograft model *in vivo* [23]. Furthermore, overexpression of EphB4 in the mammary epithelium accelerated tumor onset and lung metastasis in MMTV-Neu animals [24]. Independent studies reported EphB4 and EphB2 overexpression in human breast cancer [22], [25]. Higher EphB2 expression was associated with poor overall and disease-free survival whereas EphB4 protein expression increased with grade and stage but showed no clear association with survival. However, stronger EphB2 and EphB4 staining was reported in normal breast glandular epithelium than in tumor epithelium [22], [26], and systemic delivery of ephrin-B2-Fc inhibited the growth of MDA-MB-435 tumor xenografts via EphB4 mediated activation of the Abl/Crk pathway, which inhibits tumor cell growth and motility in breast cancer cells [27].

These observations highlight the often paradoxical findings regarding Eph RTKs in tumor promotion versus tumor suppression [28]. Our analyses of multiple large patient datasets revealed a correlation between elevated *ephB4* mRNA expression and reduced overall and recurrence-free survival. Thus, further analysis of EphB4 expression in both tumor parenchyma and the surrounding stroma is necessary, with human patient samples carefully stratified by stage and grade, as well as by molecular subtype and treatment regimen. Particular attention should be paid to expression profiles in tumor endothelium, given the role of B class receptors like EphB4 in angiogenesis and tumor neovascularization [28], as well as vessel maturation and vascular integrity [29].

While we did not observe any significant associations between EphA5 and clinical outcome, an independent profiling study reported reduced *ephA5* expression in human breast cancer samples relative to normal human breast tissue, likely due to aberrant promoter methylation [30]. While several other studies reported EphB6 promoter methylation and a tumor suppressor function [31], [32], [33], [34], [35], our data revealed a significant association between elevated *ephB6* and poorer overall and recurrence-free survival in breast cancer. The other negative associations that we observed between survival/recurrence and elevated mRNA expression of *ephA2*, *ephA4*, *ephA7*, and *ephB4* are consistent with laboratory data for some Eph family members (e.g. EphA2, EphA7), but not others (e.g. EphA4, EphB4, EphB6), in human breast cancer cell lines [31]. At least one explanation may be ligand-independent versus dependent signaling. We found an inverse correlation between EphA2 and ephrin-A1 protein expression in a significant number of invasive ductal carcinoma samples in lymph node relative to normal breast and ductal carcinomas confined to the breast, which co-express both. This observation is consistent with breast cancer cell line expression profiles [36] and with laboratory data in which ephrin-A1 ligand inhibits tumor cell growth and invasion [36], [37], [38]. We also observed, however, that co-expression of EphA2 and ephrin-A1 correlate with recurrence in Stage I prognostic TMAs. These data hightlight the importance of profiling the full spectrum of relevant ephrin-ligands as well as Eph RTKs in order to elucidate potential differences in clinical outcome associated with the presence or absence of ligand.

In summary, our analysis of expression profiles in large breast cancer datasets and in breast cancer TMAs support the clinical relevance for several Eph RTKs in human breast cancer. In addition to confirming relevance of more well-studied family members like EphA2 and EphB4, we also uncovered associations between EphA4, EphA7, EphB2, and EphB6 and overall/recurrence-free survival. The causal role of these Eph RTKs in cancer, however, must be further investigated in cell lines and animal models. Moreover, our data suggest the importance of profiling Eph family members in the context of relevant ligands and across a broad spectrum of stages in order to understand their complex roles in human cancer.

## Materials and Methods

### Reagents

Antibodies against the following proteins were used: rabbit anti-EphA2 (Life Technologies/Zymed Laboratories, Carlsbad, CA; clone 347400); rabbit anti-EphA4 (Lifespan Biosciences, Seattle, WA; clone LS-A2482; Abnova, Walnut, CA; PAB3007); rabbit anti-EphA7 (Abgent, San Diego, CA; clone RB1641/RB1642) and synthetic blocking peptide (Abgent; BP7612b); rabbit EphA8 (Abnova, PAB3015); rabbit anti-ephrin-A1 (Amgen/Immunex, Thousand Oaks, CA; clone P2); rabbit anti-EphB4 (Abgent; clone RB14731) and synthetic blocking peptide (Abgent; BP7625d); rabbit anti-EphB6 (Abgent; clone AP7627b) and synthetic blocking peptide (Abgent; BP7627b). Human breast tissue microarrays (TMA) were purchased from Cybrdi, Inc. (Rockville, MD), US Biomax (Rockville, MD), or from the National Cancer Institute Cooperative Breast Cancer Tissue Resource (NCI CBCTR). TMAs analyzed included breast carcinoma tissue array panel I with normal breast tissue controls (Cybrdi, CC08-10-001) and breast ductal carcinoma/metastasized to lymph nodes and normal breast tissue array (Cybrdi, CC08-21-002), breast disease spectrum tissue array/progression array (US Biomax, BR480), and Stage I prognostic array (CBCTR). Biotin goat anti-rabbit IgG was obtained from BD Biosciences (Pharmingen, San Diego, CA). Streptvidin horseradish peroxidase conjugate was purchased Life Technologies/Molecular Probes, and liquid 3,3′-diaminobenzidine tetrahydrochloride (DAB) substrate kit was from Life Technologies/Zymed Laboratories. Meyer's hematoxylin was purchased from Sigma-Aldrich (St. Louis, MO).

### mRNA expression profiling

Analysis of mRNA encoding human Eph RTK and ephrin gene products in the human breast cancer datasets [10], [11], [39] was performed in collaboration with the Vanderbilt-Ingram Cancer Center's Biostatistics Core Resource. Expression levels were analyzed in relation to overall and/or recurrence-free survival using Log Rank and Cox analyses.

### Immunohistochemistry

Immunohistochemical staining for Eph RTKs and ephrin-A1 was performed as described previously [14]. Briefly, sections and TMAs were deparaffinized with xylenes and rehydrated through a series of graded alcohols to PBS. The sections were subjected to thermal antigen retrieval in citrate buffer (2 mM citric acid, 10 mM sodium citrate, pH 6.0) using a PickCell Laboratories 2100 Retriever as per manufacturer's instructions. Following a brief wash in PBS, endogenous peroxidases were quenched by incubation in 3% H_2_O_2_ solution (Sigma-Aldrich) for 30 minutes. Sections were washed, incubated with general non-specific blocking solution from room temperature, and incubated overnight at 4°C with primary antibodies diluted in blocking solution. Sections were washed and incubated with biotinylated secondary antibodies for 1 hour at room temperature. Sections were incubated with diluted avidin-peroxidase reagent, washed, and stained with DAB substrate. After hematoxylin counterstain, sections were mounted and photographed on an Olympus BX60 microscope using a digital camera and NIH Scion Image software.

Where indicated, diluted antibodies were pre-incubated with 1 µg of competitor peptide for 1 hour at 4°C, rotating, prior to incubation with tissue sections to control for specificity. Additional controls included normal mouse kidney, liver, and small intestine. For EphA2 and ephrin-A1, tissue from deficient mouse models [18], [40] was probed to confirm specificity of staining.

### Scoring relative Eph and ephrin expression levels in TMAs

For commercial TMAs, relative expression was scored using a continuous scale as follows: 0 = 0–10% positive tumor epithelium, 1 = 10–25% positive tumor epithelium, 2 = 25–50% positive tumor epithelium, and 3 = >50% positive tumor epithelium/core. TMA cores were scored blind by three independent individuals, the average of which was reported here. Differential expression between tissue samples were quantified and statistically analyzed using Chi square analysis.

For TMAs purchased from the NCI CBCTR, stained cores were scanned and staining quantified using the Ariol SL-50 platform through the Vanderbilt University Epithelial Biology Center (EBC). Stained cores were scanned and areas encompassing tumor epithelium were selected for computer-based calculation of the percentage positive tumor epithelium (DAB stained) relative to total tumor epithelium (hematoxylin stained) with a scale for relative intensity.

### Statistical Analyses

Statistical analyses were performed in collaboration with the Vanderbilt-Ingram Cancer Center's Biostatistics Core Resource using Software: R2.12.1 [http://www.r-project.org/; [41]]. The survival curves from Kaplan-Meier was created and plotted by the function “survfit” under R package “survival.” P values shown on KM plots were calculated based on log rank test between two survival curves of high or low expression groups [42]. The c-indexes shown on KM plots were calculated by the function “rcorr.cens” under R package “Hmisc” [43].

The association between individual gene expression level and clinical endpoint (overall survival, metastasis-free survival, and recurrence survival were analyzed with the use of a Cox proportional hazard (PH) model. The function “coxph” in R package “survival” was applied [44]. For analysis of ephrin-A1 and EphA2 protein expression in NCI CBCTR TMAs, we used Pearson's product moment correlation coefficient to test for association between paired samples. The function “cor.test” in R package “stats” was employed.

To rule out any potential over-fitting problem of the identified correlation between EphA2 and ephrin-A1 co-expression and recurrence in the Stage I prognostic TMAs, we used bootstrap based correlation analysis. The “sample” method in R package “stat” is used perform the bootstrap analysis [45], [46]. We randomly selected (with replacement) a sample size of 72 from 446 non-recurrent patients 10,000 times.

## Supporting Information

Figure S1 **RNA expression of EphA2, EphA4, and EphA7 negatively correlates with recurrence-free survival in human breast cancer.** Kaplan-Meier kinetic analyses of the van der Vijver dataset, with microarray profiles of 295 human breast tumors and associated clinical data. The impact of elevated *ephA2* (A), *ephA4* (B), and *ephA7* (C), expression on recurrence-free survival was analyzed by Log-Rank tests.
(TIF)

Figure S2 **RNA expression of EphB4 and EphB6 negatively correlates with recurrence-free survival in human breast cancer.** Kaplan-Meier kinetic analyses of the van der Vijver dataset, with microarray profiles of 295 human breast tumors and associated clinical data. The impact of elevated *ephB4* (A) and *ephB6* (B) expression on recurrence-free survival was analyzed by Log-Rank tests.
(TIF)

Figure S3 **RNA expression of EphA2, EphA4, and EphA7 negatively correlates with metastasis-free survival in human breast cancer.** Kaplan-Meier kinetic analyses of the Veer, with microarray profiles of 117 human breast tumors and associated clinical data. The impact of elevated *ephA2* (A), *ephA4* (B), and *ephA7* (C), expression on metastasis-free survival was analyzed by Log-Rank tests.
(TIF)

Figure S4 **RNA expression of EphB4 and EphB6 negatively correlates with metastasis-free survival in human breast cancer.** Kaplan-Meier kinetic analyses of the Veer dataset, with microarray profiles of 117 human breast tumors and associated clinical data. The impact of elevated *ephB4* (A) and *ephB6* (B) expression on metastasis-free survival was analyzed by Log-Rank tests.
(TIF)

Figure S5 **RNA expression of ER and PR correlates with overall and metastasis-free survival in human breast cancer, validating microarray datasets.** Kaplan-Meier kinetic analyses of the van der Vijver and Veer datasets, with microarray profiles of 295 and 117 human breast tumors and associated clinical data, respectively. The impact of elevated estrogen receptor *ESR1* (A, C) and progesterone receptor *PGR* (B, D) expression on overall and metastasis-free survival was analyzed by Log-Rank tests.
(TIF)

Figure S6 **RNA and protein expression of EphA8 in human breast cancer.** Kaplan-Meier kinetic analyses of the van der Vijver (A) and Veer (B) datasets, with microarray profiles of 295 and 117 human breast tumors and associated clinical data, respectively. The impact of *ephA8* expression on overall, recurrence-free, and metastasis-free survival was analyzed by Log-Rank tests. (C) Immunohistochemical analysis of human breast tissue microarrays (TMAs) was performed to compare relative expression in normal/benign epithelium (n = 8 samples) versus invasive ductal carcinoma (n = 126 samples) for EphB4 (A) and EphB6 (B). Arrows indicate tumor epithelium in photomicrographs. Scale bar = 50 µm. No statistically significant correlations between RNA expression/clinical outcome or protein expression/malignancy were observed.
(TIF)

Figure S7 **Anti-Eph and ephrin antibody validation.** Immunohistochemical analysis of mouse mammary tissue from wild-type (A, E), EphA2-deficient (A), or ephrin-A1-deficient (E) was performed to validate specificity of anti-EphA2 and anti-ephrin-A1 antibodies. Arrows indicate mammary/tumor epithelium in photomicrographs. Scale bar = 50 µm. Immunohistochemical analysis of mouse kidney tissue was performed to validate specificity of anti-EphA4 (B), anti-EphA7 (C), anti-EphA8 (D), anti-EphB4 (F), and anti-EphB6 antibodies. We compared staining in the presence or absence of competitor peptides that were pre-incubated with primary antibodies. Arrowheads indicate distal tubules.
(TIF)

Table S1 Association between Eph receptor expression and metastasis-free survival in human breast cancer.
(DOCX)

## References

[1] Michael S, Charikleia S, Konstantinos K (2011). Lymphedema and breast cancer: a review of the literature.. Breast Cancer.

[2] Azim HA, de Azambuja E, Colozza M, Bines J, Piccart MJ (2011). Long-term toxic effects of adjuvant chemotherapy in breast cancer.. Ann Oncol.

[3] Stewart FA, Hoving S, Russell NS (2010). Vascular Damage as an Underlying Mechanism of Cardiac and Cerebral Toxicity in Irradiated Cancer Patients.. Radiat Res.

[4] Tonezzer T, Pereira CM, Filho UP, Marx A (2010). Hormone therapy/adjuvant chemotherapy induced deleterious effects on the bone mass of breast cancer patients and the intervention of physiotherapy: a literature review.. Eur J Gynaecol Oncol.

[5] Pasquale EB (2010). Eph receptors and ephrins in cancer: bidirectional signalling and beyond.. Nat Rev Cancer.

[6] Brantley-Sieders DM, Chen J (2004). Eph receptor tyrosine kinases in angiogenesis: from development to disease.. Angiogenesis.

[7] Brantley-Sieders D, Schmidt S, Parker M, Chen J (2004). Eph receptor tyrosine kinases in tumor and tumor microenvironment.. Curr Pharm Des.

[8] Vaught D, Brantley-Sieders DM, Chen J (2008). Eph receptors in breast cancer: roles in tumor promotion and tumor suppression.. Breast Cancer Res.

[9] Lackmann M, Boyd AW (2008). Eph, a protein family coming of age: more confusion, insight, or complexity?. Sci Signal.

[10] van de Vijver MJ, He YD, van't Veer LJ, Dai H, Hart AA (2002). A gene-expression signature as a predictor of survival in breast cancer.. N Engl J Med.

[11] van 't Veer LJ, Dai H, van de Vijver MJ, He YD, Hart AA (2002). Gene expression profiling predicts clinical outcome of breast cancer.. Nature.

[12] Vaught D, Chen J, Brantley-Sieders DM (2009). Regulation of mammary gland branching morphogenesis by EphA2 receptor tyrosine kinase.. Mol Biol Cell.

[13] Kouros-Mehr H, Werb Z (2006). Candidate regulators of mammary branching morphogenesis identified by genome-wide transcript analysis.. Dev Dyn.

[14] Brantley DM, Cheng N, Thompson EJ, Lin Q, Brekken RA (2002). Soluble Eph A receptors inhibit tumor angiogenesis and progression in vivo.. Oncogene.

[15] Ogawa K, Pasqualini R, Lindberg RA, Kain R, Freeman AL (2000). The ephrin-A1 ligand and its receptor, EphA2, are expressed during tumor neovascularization.. Oncogene.

[16] Zelinski DP, Zantek ND, Stewart JC, Irizarry AR, Kinch MS (2001). EphA2 overexpression causes tumorigenesis of mammary epithelial cells.. Cancer Res.

[17] Pan M (2005). Overexpression of *EphA2* gene in invasive human breast cancer and its association with hormone receptor status.. J of Clin Oncol [Meeting Abstracts].

[18] Brantley-Sieders DM, Zhuang G, Hicks D, Fang WB, Hwang Y (2008). The receptor tyrosine kinase EphA2 promotes mammary adenocarcinoma tumorigenesis and metastatic progression in mice by amplifying ErbB2 signaling.. J Clin Invest.

[19] Fang WB, Brantley-Sieders DM, Parker MA, Reith AD, Chen J (2005). A kinase-dependent role for EphA2 receptor in promoting tumor growth and metastasis.. Oncogene.

[20] Zhuang G, Brantley-Sieders DM, Vaught D, Yu J, Xie L (2010). Elevation of receptor tyrosine kinase EphA2 mediates resistance to trastuzumab therapy.. Cancer Res.

[21] Gokmen-Polar Y, Toroni RA, Hocevar BA, Badve S, Zhao Q (2010). Dual targeting of EphA2 and ER restores tamoxifen sensitivity in ER/EphA2-positive breast cancer.. Breast Cancer Res Treat.

[22] Wu Q, Suo Z, Risberg B, Karlsson MG, Villman K (2004). Expression of Ephb2 and Ephb4 in breast carcinoma.. Pathol Oncol Res.

[23] Kumar SR, Singh J, Xia G, Krasnoperov V, Hassanieh L (2006). Receptor tyrosine kinase EphB4 is a survival factor in breast cancer.. Am J Pathol.

[24] Munarini N, Jager R, Abderhalden S, Zuercher G, Rohrbach V (2002). Altered mammary epithelial development, pattern formation and involution in transgenic mice expressing the EphB4 receptor tyrosine kinase.. J Cell Sci.

[25] Berclaz G, Andres AC, Albrecht D, Dreher E, Ziemiecki A (1996). Expression of the receptor protein tyrosine kinase myk-1/htk in normal and malignant mammary epithelium.. Biochem Biophys Res Commun.

[26] Berclaz G, Flutsch B, Altermatt HJ, Rohrbach V, Djonov V (2002). Loss of EphB4 receptor tyrosine kinase protein expression during carcinogenesis of the human breast.. Oncol Rep.

[27] Noren NK, Foos G, Hauser CA, Pasquale EB (2006). The EphB4 receptor suppresses breast cancer cell tumorigenicity through an Abl-Crk pathway.. Nat Cell Biol.

[28] Noren NK, Pasquale EB (2007). Paradoxes of the EphB4 receptor in cancer.. Cancer Res.

[29] Salvucci O, Maric D, Economopoulou M, Sakakibara S, Merlin S (2009). EphrinB reverse signaling contributes to endothelial and mural cell assembly into vascular structures.. Blood.

[30] Fu DY, Wang ZM, Wang BL, Chen L, Yang WT (2010). Frequent epigenetic inactivation of the receptor tyrosine kinase EphA5 by promoter methylation in human breast cancer.. Hum Pathol.

[31] Fox BP, Kandpal RP (2004). Invasiveness of breast carcinoma cells and transcript profile: Eph receptors and ephrin ligands as molecular markers of potential diagnostic and prognostic application.. Biochem Biophys Res Commun.

[32] Fox BP, Tabone CJ, Kandpal RP (2006). Potential clinical relevance of Eph receptors and ephrin ligands expressed in prostate carcinoma cell lines.. Biochem Biophys Res Commun.

[33] Fox BP, Kandpal RP (2009). EphB6 receptor significantly alters invasiveness and other phenotypic characteristics of human breast carcinoma cells.. Oncogene.

[34] Fox BP, Kandpal RP (2010). DNA-based assay for EPHB6 expression in breast carcinoma cells as a potential diagnostic test for detecting tumor cells in circulation.. Cancer Genomics Proteomics.

[35] Truitt L, Freywald T, DeCoteau J, Sharfe N, Freywald A (2010). The EphB6 receptor cooperates with c-Cbl to regulate the behavior of breast cancer cells.. Cancer Res.

[36] Macrae M, Neve RM, Rodriguez-Viciana P, Haqq C, Yeh J (2005). A conditional feedback loop regulates Ras activity through EphA2.. Cancer Cell.

[37] Miao H, Wei BR, Peehl DM, Li Q, Alexandrou T (2001). Activation of EphA receptor tyrosine kinase inhibits the Ras/MAPK pathway.. Nat Cell Biol.

[38] Guo H, Miao H, Gerber L, Singh J, Denning MF (2006). Disruption of EphA2 receptor tyrosine kinase leads to increased susceptibility to carcinogenesis in mouse skin.. Cancer Res.

[39] Nakamura R, Kataoka H, Sato N, Kanamori M, Ihara M (2005). EPHA2/EFNA1 expression in human gastric cancer.. Cancer Sci.

[40] Frieden LA, Townsend TA, Vaught DB, Delaughter DM, Hwang Y (2010). Regulation of heart valve morphogenesis by Eph receptor ligand, ephrin-A1.. Dev Dyn.

[41] Team RDC (2010). R: A language and environment for statistical computing.

[42] Harrington DP, Fleming TR (1982). A class of rank test procedures for censored survival data.. Biometrika.

[43] Newson R (2006). Confidence intervals for rank statistics: Somers' D and extensions.. The Stata Journal.

[44] Therneau TM, Grambsch PM, Dietz K, Gail M, Krickeberg K, Samet J, Tsiatis A (2000). Modeling survival data: extending the Cox model;.

[45] Ripley BD (1987). Stochastic Simulation.

[46] Becker RA, Chambers JM, Wilks AR, Brookscole Wa (1998). The New S Language: A Programming Environment for Data Analysis and Graphics;.

