# Supplementary figures and images for: Eph/Ephrin Profiling in Human Breast Cancer Reveals Significant Associations between Expression Level and Clinical Outcome
Source: PLoS One. 2011 Sep 15;6(9):e24426. doi: 10.1371/journal.pone.0024426 (PMC3174170; doi:10.1371/journal.pone.0024426)

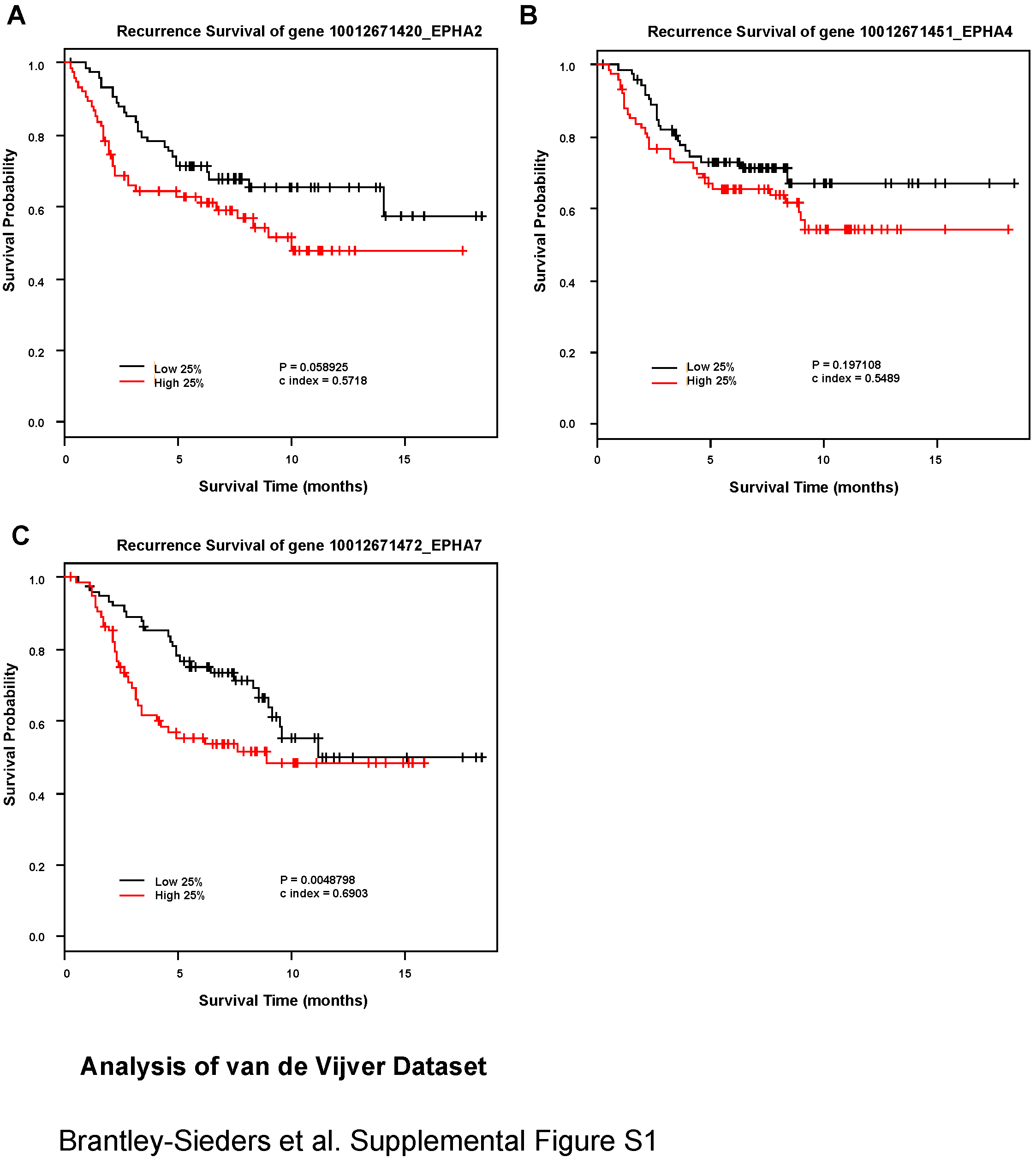

Supplement: Figure S1 — RNA expression of EphA2, EphA4, and EphA7 negatively correlates with recurrence-free survival in human breast cancer. Kaplan-Meier kinetic analyses of the van der Vijver dataset, with microarray profiles of 295 human breast tumors and associated clinical data. The impact of elevated ephA2 (A), ephA4 (B), and ephA7 (C), expression on recurrence-free survival was analyzed by Log-Rank tests. (TIF) [file pone.0024426.s001.tif]

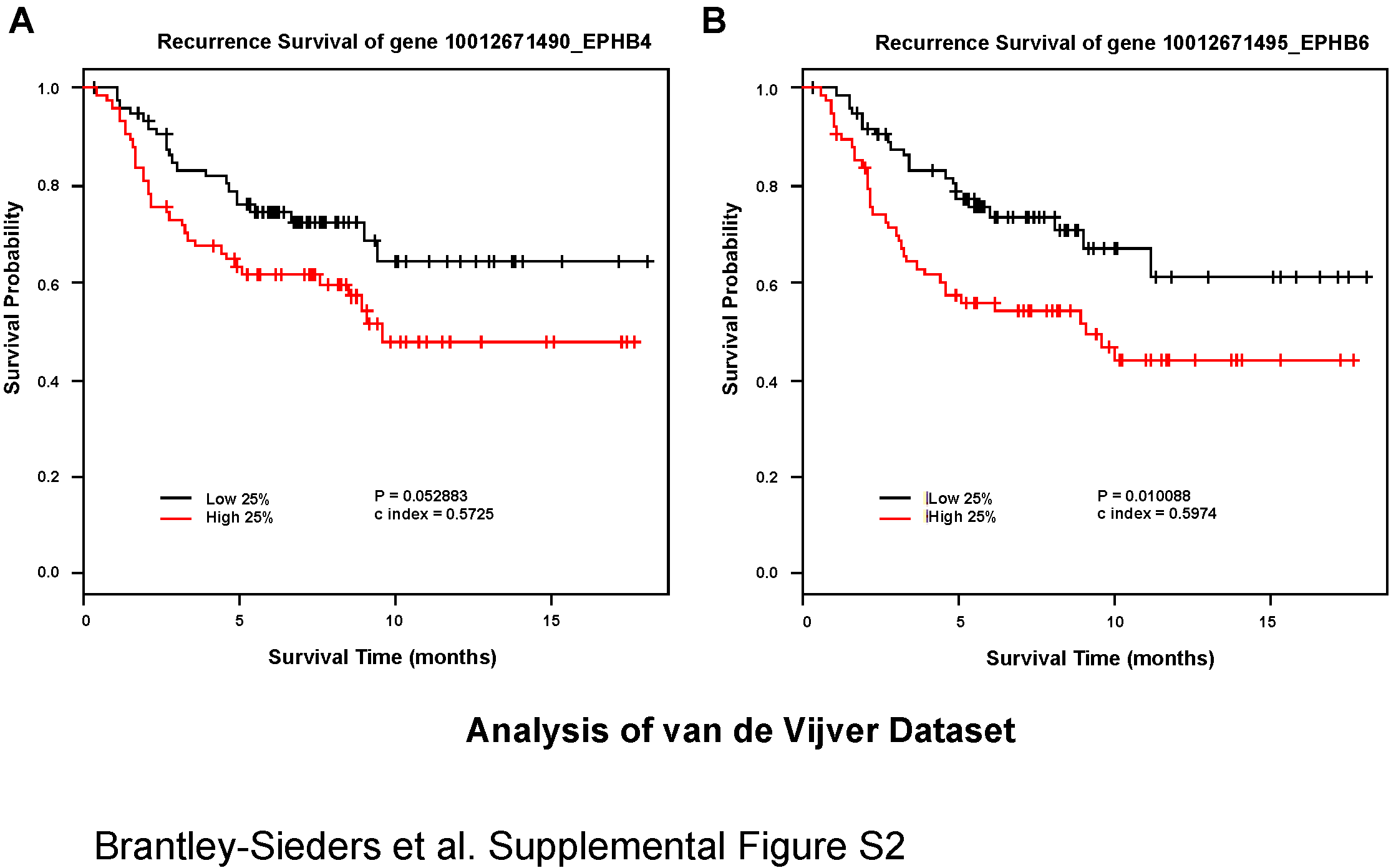

Supplement: Figure S2 — RNA expression of EphB4 and EphB6 negatively correlates with recurrence-free survival in human breast cancer. Kaplan-Meier kinetic analyses of the van der Vijver dataset, with microarray profiles of 295 human breast tumors and associated clinical data. The impact of elevated ephB4 (A) and ephB6 (B) expression on recurrence-free survival was analyzed by Log-Rank tests. (TIF) [file pone.0024426.s002.tif]

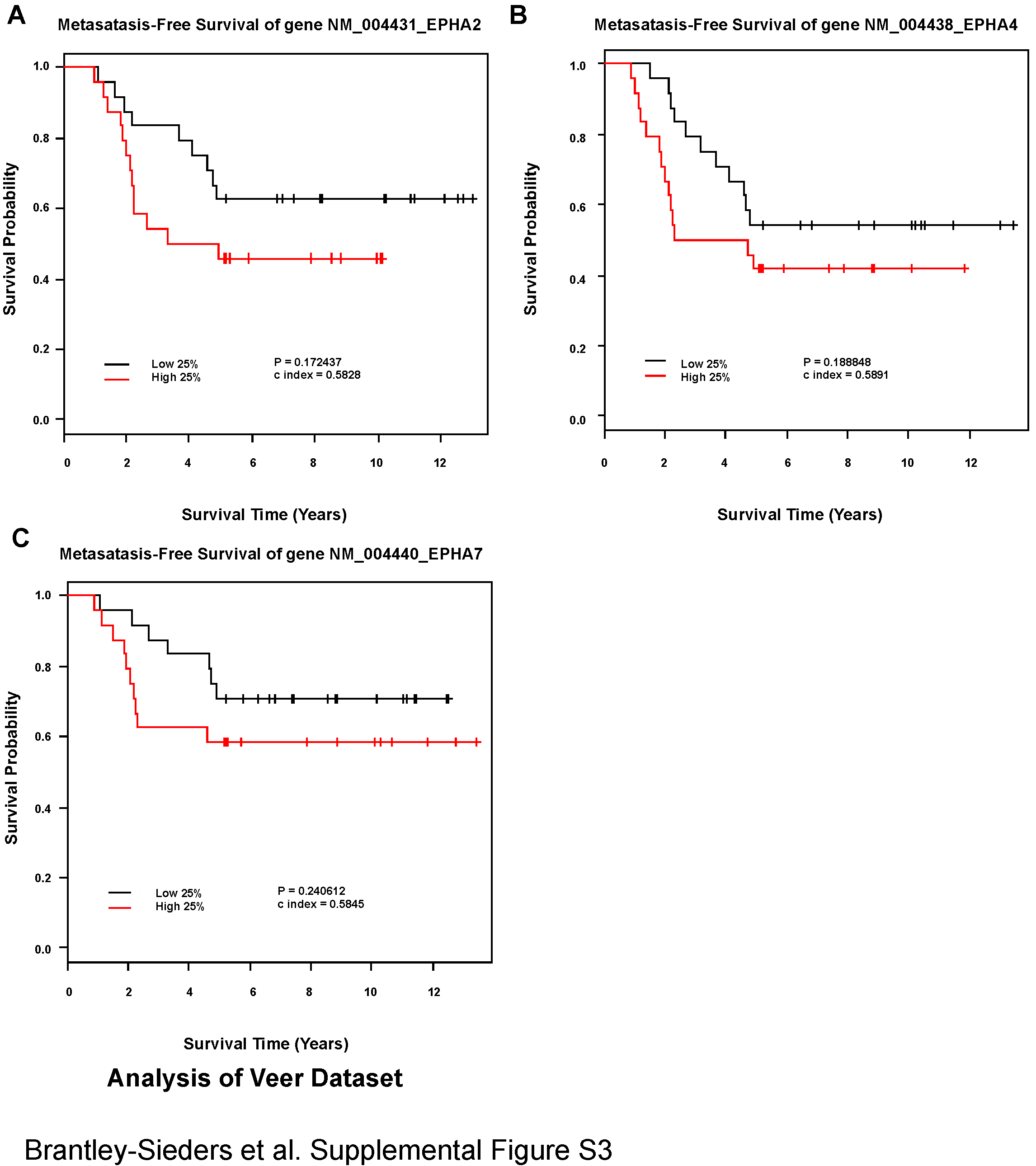

Supplement: Figure S3 — RNA expression of EphA2, EphA4, and EphA7 negatively correlates with metastasis-free survival in human breast cancer. Kaplan-Meier kinetic analyses of the Veer, with microarray profiles of 117 human breast tumors and associated clinical data. The impact of elevated ephA2 (A), ephA4 (B), and ephA7 (C), expression on metastasis-free survival was analyzed by Log-Rank tests. (TIF) [file pone.0024426.s003.tif]

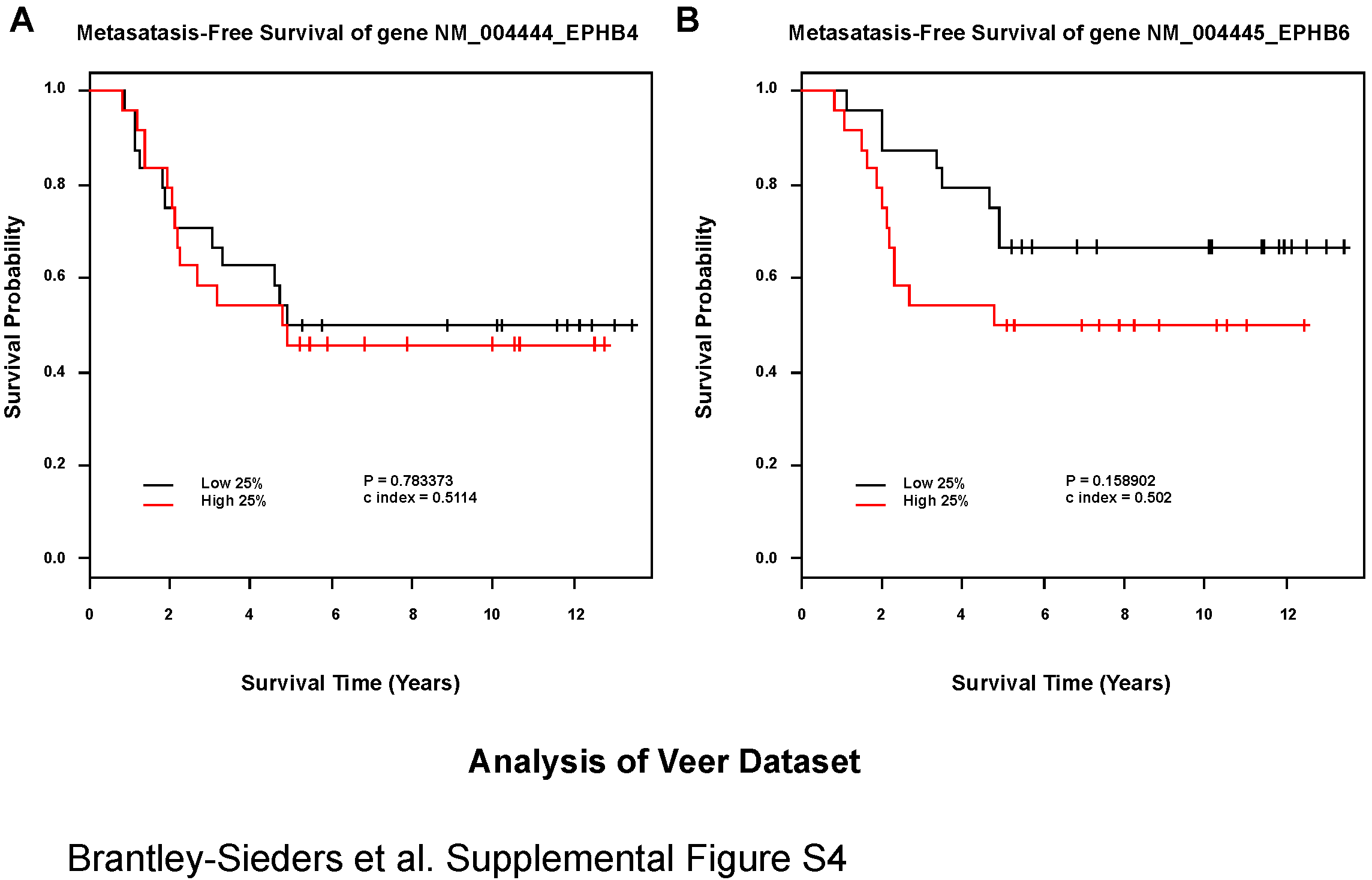

Supplement: Figure S4 — RNA expression of EphB4 and EphB6 negatively correlates with metastasis-free survival in human breast cancer. Kaplan-Meier kinetic analyses of the Veer dataset, with microarray profiles of 117 human breast tumors and associated clinical data. The impact of elevated ephB4 (A) and ephB6 (B) expression on metastasis-free survival was analyzed by Log-Rank tests. (TIF) [file pone.0024426.s004.tif]

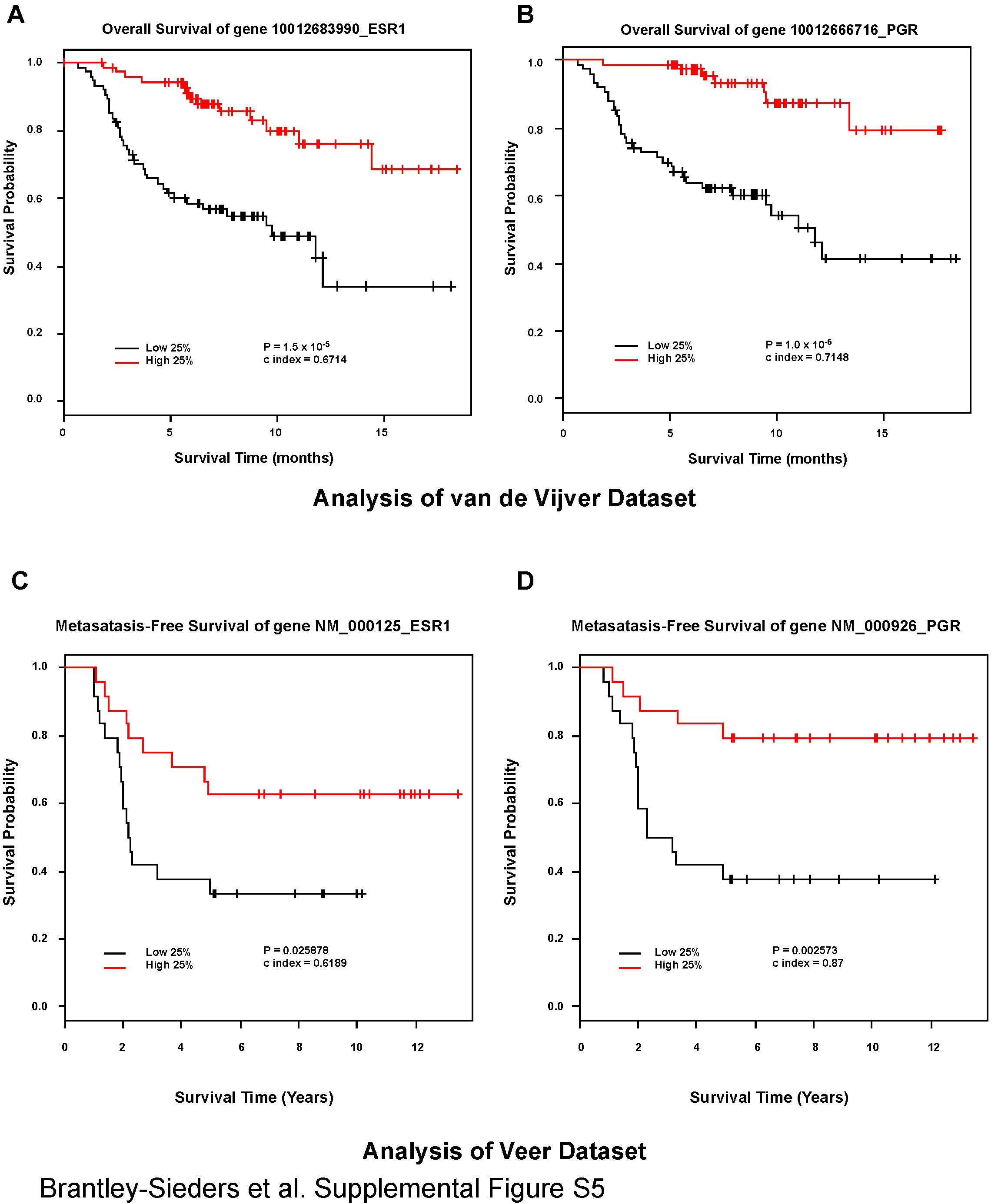

Supplement: Figure S5 — RNA expression of ER and PR correlates with overall and metastasis-free survival in human breast cancer, validating microarray datasets. Kaplan-Meier kinetic analyses of the van der Vijver and Veer datasets, with microarray profiles of 295 and 117 human breast tumors and associated clinical data, respectively. The impact of elevated estrogen receptor ESR1 (A, C) and progesterone receptor PGR (B, D) expression on overall and metastasis-free survival was analyzed by Log-Rank tests. (TIF) [file pone.0024426.s005.tif]

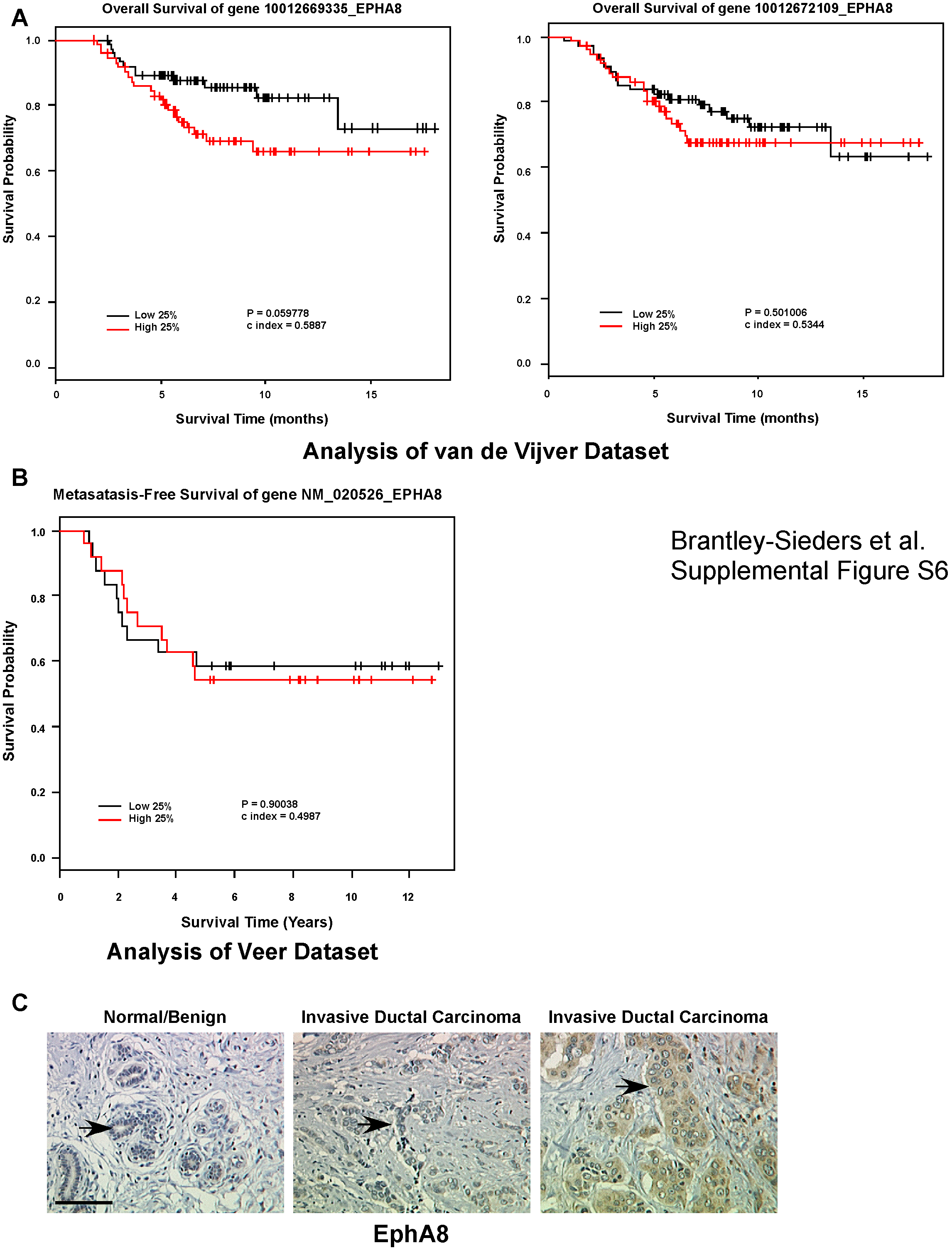

Supplement: Figure S6 — RNA and protein expression of EphA8 in human breast cancer. Kaplan-Meier kinetic analyses of the van der Vijver (A) and Veer (B) datasets, with microarray profiles of 295 and 117 human breast tumors and associated clinical data, respectively. The impact of ephA8 expression on overall, recurrence-free, and metastasis-free survival was analyzed by Log-Rank tests. (C) Immunohistochemical analysis of human breast tissue microarrays (TMAs) was performed to compare relative expression in normal/benign epithelium (n = 8 samples) versus invasive ductal carcinoma (n = 126 samples) for EphB4 (A) and EphB6 (B). Arrows indicate tumor epithelium in photomicrographs. Scale bar = 50 µm. No statistically significant correlations between RNA expression/clinical outcome or protein expression/malignancy were observed. (TIF) [file pone.0024426.s006.tif]

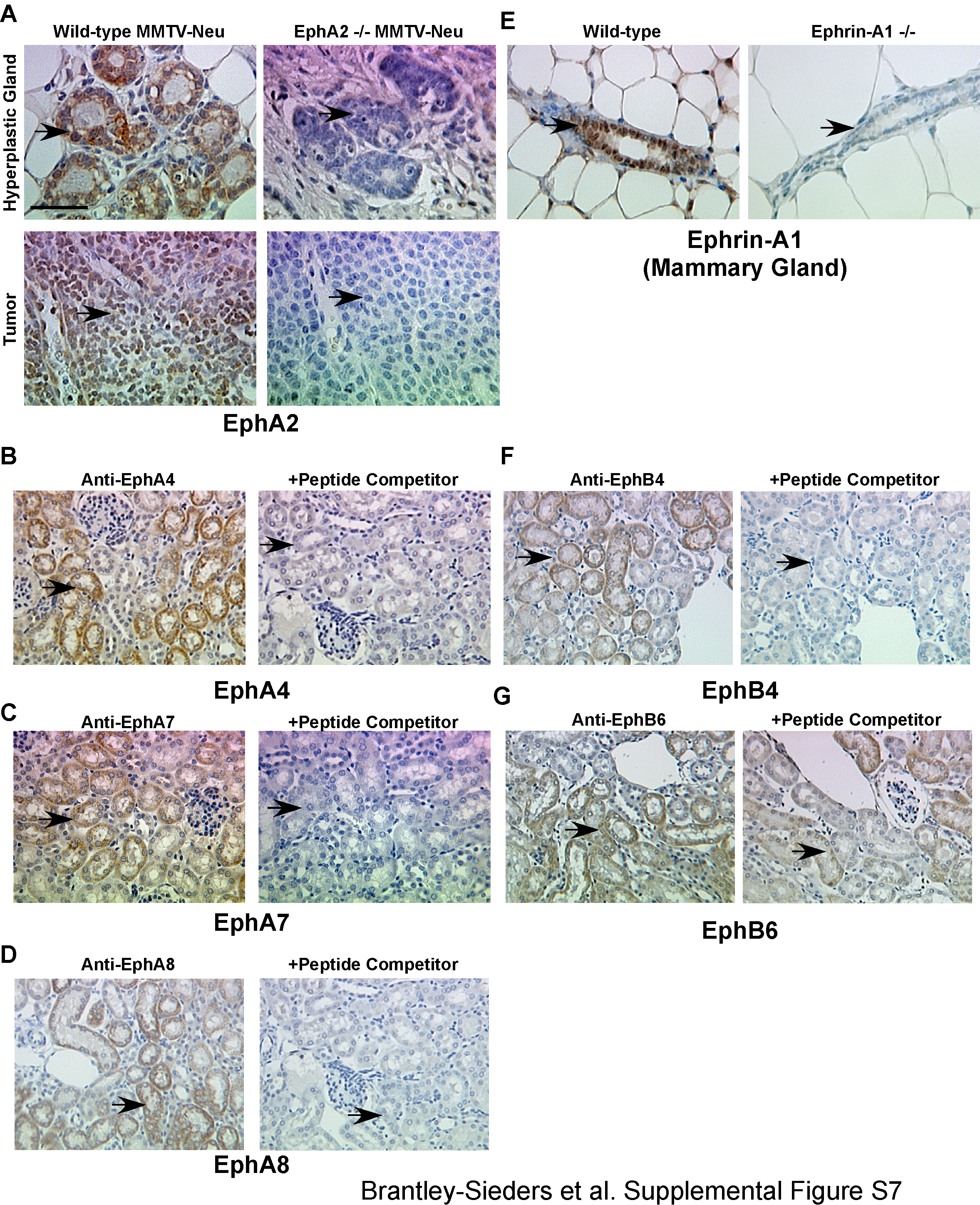

Supplement: Figure S7 — Anti-Eph and ephrin antibody validation. Immunohistochemical analysis of mouse mammary tissue from wild-type (A, E), EphA2-deficient (A), or ephrin-A1-deficient (E) was performed to validate specificity of anti-EphA2 and anti-ephrin-A1 antibodies. Arrows indicate mammary/tumor epithelium in photomicrographs. Scale bar = 50 µm. Immunohistochemical analysis of mouse kidney tissue was performed to validate specificity of anti-EphA4 (B), anti-EphA7 (C), anti-EphA8 (D), anti-EphB4 (F), and anti-EphB6 antibodies. We compared staining in the presence or absence of competitor peptides that were pre-incubated with primary antibodies. Arrowheads indicate distal tubules. (TIF) [file pone.0024426.s007.tif]
