# Supplementary material for: Eph/Ephrin Profiling in Human Breast Cancer Reveals Significant Associations between Expression Level and Clinical Outcome
Source: PLoS One. 2011 Sep 15;6(9):e24426. doi: 10.1371/journal.pone.0024426 (PMC3174170; doi:10.1371/journal.pone.0024426)
Supplement: Table S1 — Association between Eph receptor expression and metastasis-free survival in human breast cancer. (DOCX) [file pone.0024426.s008.docx]

Table S1. Association between Eph receptor expression and metastasis-free survival in human breast cancer.

| **Eph Receptor** | **RR Metastasis-Free Survival (95% CI)** | **P-Value** |
| --- | --- | --- |
| EphA2 | 5.60 (1.41-22.3) | 0.0146* |
| EphA4 | 2.42 (0.59-9.93) | 0.2198 |
| EphA7 | 1.01 (0.33-2.98) | 0.9817 |
|  |  |  |
| EphB4 | 3.06 (0.21-45.4) | 0.4159 |

Cox Model Analysis: Relative risk (RR) associated with elevated Eph receptor molecule expression and metastasis-free survival in the Veer dataset. *Statistically significant association.
